# Spatial matching on the urban labor market: estimates with unique micro data

**DOI:** 10.1186/s12651-021-00293-1

**Published:** 2021-04-06

**Authors:** Marcin Wozniak

**Affiliations:** Faculty of Human Geography and Planning, Adam Mickiewicz University, Poznan, Poland

**Keywords:** Spatial matching function, Spatial correlation, Urban labor market, J23, J64, R12, E24

## Abstract

In the paper, we investigate spatial relationship on the labor market of Poznań agglomeration (Poland) with unique data on job vacancies. We have developed spatial panel models to assess the search and matching process with a particular focus on spatial spillovers. In general, spatial models may provide different findings than regular panel models regarding returns to scale in matching technology. Moreover, we have identified global spillover effects as well as other factors that impact the job-worker matching. We underline the role of data on job vacancies: the data retrieved from commercial job portals produced much more reliable estimates than underestimated registered data.

## Introduction

Labor markets are spatial in their nature. In metropolitan areas, people commute to achieve a better-paid or more satisfactory job. Large cities are surrounded by minor counties the function of which is often to provide shelter and basic services to people who work in the city and spend there most of their daytime. However, these surrounding counties are not homogenous, and may strongly differ in their socio-economic development and functions. They formed more or less self-sufficient enclaves that are somehow dependent on its large metropolitan neighbor.

The aim of this paper is to investigate spatial relationships on the urban labor market with two concepts: spatial panel matching function models and in-depth spatial correlation analysis. In the research we employed unique data on job vacancies derived directly from the most substantial Polish internet job portals. We focus on the Poznań agglomeration (Poland)—one of the largest metropolitan areas in Poland that consists of 18 counties (LAU level 2). LAU2 (formerly NUTS5) is the smallest administrative unit to divide the territory of the European Union. It includes municipalities, agglomerations or communes (Eurostat).

**Fig. 1:**
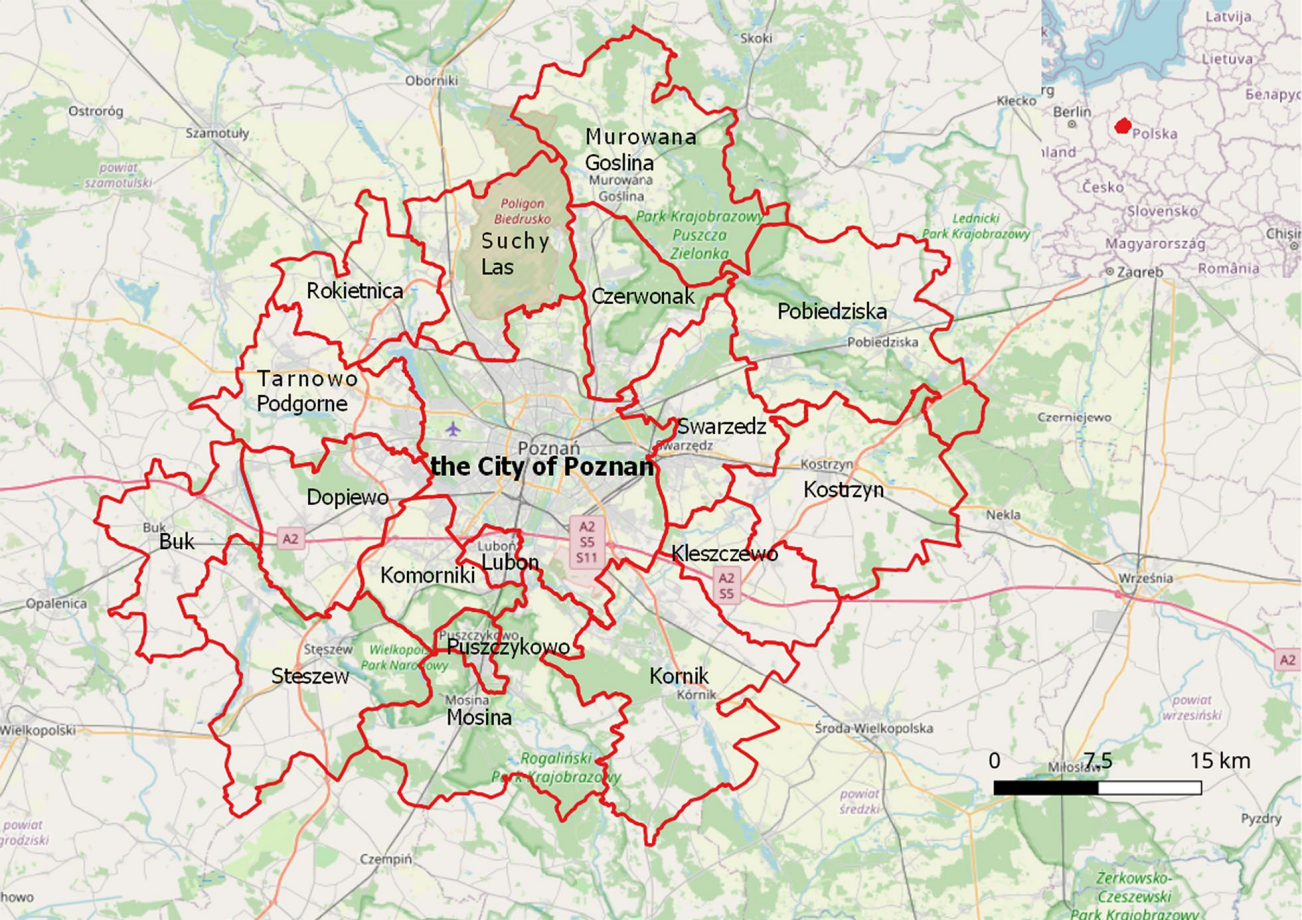
Poznań agglomeration (the city of Poznań and 17 neighboring counties) (Source: own elaborations on a basis of OpenStreetMap)

The Poznań agglomeration is one of the largest metropolitan areas in Poland and lies in the east–west line in central west Poland. It is also a workhorse of a regional economy with the dominant industries of construction, services, trade, and logistics. The Poznań agglomeration is also a highly international place with the Amazon distribution warehouse, Volkswagen Factory, Carlsberg, Bertelsmann, among many others. Poznań has a record-breaking low-level of the unemployment rate that dropped to 1.1% in December 2019. As a result, one of the major problems of local employers was worker shortages (Manpower Group 2020). The distance from the central city to the boundaries of the agglomeration does not exceed 25 km. The metropolitan area is connected with the surrounding counties through an extensive railway and bus network. This situation is beneficial to commuters which can travel relatively easy to their workplaces (Bul 2014). It is also worth noting that despite the undoubtful economic domination of the core unit, some small but strong local economic centers can also be pointed out. This is because many large enterprises are located outside the metropolitan area (e.g. in Tarnowo Podgórne, Suchy Las, Kostrzyn, Swarzędz, Czerwonak, Komorniki). Figure 1 presents the geographical location of Poznań and the surrounding counties (Poznań agglomeration).

The general theoretical foundations of equilibrium models of spatial labor market matching were laid by Rouwedall (1998) who exceeded the standard sequential job search model (Mortensen 1986) by commuting behavior patterns between residential and employment locations. In the model, an urbanized region with several employment centers was developed. The author proved that spatial relationships affect the behavior of job seekers in terms of excess commuting.

Among the most recent papers some general but wide insight into spatial labor market matching was done by Brancaccio et al. (2019) or Wong (2015) in a more modest version. The authors provide some general guidelines to estimating matching functions in several spatial contexts excluding the labor market (e.g. taxis, bike-sharing schemes or shipping). They focus on theoretical assumptions, parametrization and estimation procedures in the case of a Cobb–Douglas matching function with constant returns to scale. The authors underline the universality of the approach and briefly discuss the applications of the mechanism.

Some of the more specific works on labor market matching center on Germany and consider various levels of spatial aggregation. Among the most influential research is the paper by Kosfeld (2007). The author focused on German regions and proved that matching function coefficients estimations are not stable over space. In that case, the larger coefficient of a spatial lag in a given region is connected with higher mobility. Among later studies, the paper by Lottmann (2012) needs to be mentioned. The author applies the spatial matching function to the German labor market, makes use of data from 176 labor offices, and provides evidence for spatial dependencies that affect the matching process on German labor markets. The results suggest significant spatial spillovers. This means that regional policy activities have consequences on a wider scale. Other comprehensive study of the German labor markets was done by Haller and Heuermann (2016). They estimate a regional matching function on NUTS3 geographical units and prove positive and significant effects of vacancies on matching. Other similar works that investigate the spatial process on the labor market are those by Fahr and Sunde (2006) for West Germany or Hujer et al. (2009) for the estimation of the effects of labor market programs.

Among other countries of focus, one of the most interesting research is that of Manning and Petrolongo (2017). They provide a framework for job search behavior across the markets with a very large number of segments. In fact, the authors used census data on unemployment and vacancies to estimate the matching function across over 8000 wards of UK and Wales. They found that the cost connected with the distance of vacancy was relatively high. They also observed that workers were discouraged from applying for a job if they expected strong competition. Moreover, local policies stimulate the outflow from unemployment but, due to overlapping effects these programs seem ineffective.

It is worth mentioning some papers that focus on central and eastern Europe and investigate spatial matching on labor markets. The first is Burda and Profit (1996) who estimate the matching function with a spatial component. Their major finding is that the matching process in a given spatial unit is affected by neighborhoods. The closer the neighborhood the more significant the impact. Among later papers, Dmitrijeva (2008) estimates three specifications of the matching function: a stock-stock matching function, a stock-flow matching function and a spatially augmented stock-flow matching function. The author uses a data on Latvian, Slovenian and Estonian regions to study the search and match process on labor markets. Spatial spillovers exist and are statistically significant in the case of Latvia and Slovenia (estimations for Estonia were not possible due to the structure of available data.

As for Poland, the only paper is Antczak et al. (2018). The study provides the comprehensive analysis of labor markets at the LAU-1 (NUTS4) level. The authors developed models of spatial econometrics based on three different specifications of a matching function to investigate how spatial interactions affect the process of matching. They found the evidence of heterogeneity as well as clustering and polarization processes. The authors argue that the role of spatial dependencies in creating an outflow to employment is significant and underline the role of policy measures at regional level.

To our best knowledge, the paper presented is the first that deals with the spatial matching function estimation using internet data on vacancies. In addition, we did not find studies on spatial correlation at the low level of spatial aggregation (LAU2) within the EU countries. It is worth mentioning that numerous studies investigate the problem of spatial correlation and the matching process on the labor market. However, the first papers focused mainly on NUTS2 and NUTS4 areas within the EU countries. As the repercussion of this research, the general relevance of spatial interaction was confirmed for several countries, e.g. Spain (López-Tamayo et al. 2000), Finland (Ahtonen 2005), UK, Japan (Kano and Ohta 2005) or USA (Brueckner and Zenou 2003 among many others).

The contribution of the paper is therefore twofold. Firstly, we provide a supplementary insight into the research on urban labor markets at the low level of spatial aggregation (LAU2). Such studies are ultimately rare (excluding the paper by Manning and Petrolongo (2017), we did not find any contributions). Secondly, we used a unique microdata set extracted with the developed Web Application Programming Interface^1^ (API) script from popular internet job portals and Public Employment Service statistics (PES). The approach is in contrast to the majority of studies that utilize only PES that is highly underestimated in terms of vacancies (e.g. Feng and Hu 2013, Gałecka-Burdziak 2017). Therefore, our data provide a reliable insight into the supply and demand of the local labor market in each of its spatial units and constitute the basis for the analysis. As a result, we extend the knowledge in these fields by adding the in-depth analysis of the urban labor market.

## Public data bias

The analysis of supply and demand on the labor market is one of the main research fields in labor economics. Although the recent theories of the labor market accept the heterogeneity of jobs and workers, they also pay attention to very different details of job/employee matching processes. Search theory of labor markets and a derivative matching function concept underline a complex and time-consuming seeking procedure between employers and unemployed persons. (e.g. Rogerson et al. 2005). The crucial from the point of the matching function estimation is the quality of data. In all reported studies data are obtained from public employment services databases. However, public statistics methodology often assume that companies report the number of job vacancies voluntarily.^2^ As a result, the most frequent bias in registered data is connected with its underestimation (e.g. Paull 2002, Feng and Hu 2013, Gałecka-Burdziak 2016—for the Polish case). Additionally, for such reasons as saving time, money or just too much effort, data are usually reported with a frequency of a month, a quarter or even a year (Scott and Varian 2014). Other common problems result from a delay in obtaining the data. We get job vacancies statistics every month but in fact, we do not know what is happening between these time points. The situation in Poland is unfavorable as Public Employment Service presents only stock data at the end of each month. Some of these job offers were reported a long time ago but because of high turnover or other issues they are available in registry for months. Such statistics may be perceived as obsolete and not well suited to the reality of a highly dynamic labor market. The last but not least is a bias associated with a specific type of job vacancies that are registered in labor offices. The jobs are often addressed to low-skilled workers and the wage offer is usually equal or slightly higher than the minimum wage in Poland (Roszkowska et al. 2017). Sztandar-Sztanderska (2017) on the basis of qualitative research claims that PES in Poland is marked with institutional and organizational instability, limited human resources, insufficient spending on labor market programs. This state of affairs discourages employers from contacting labor offices and register the vacancies through the complicated, time-consuming procedure. Because of that, the number of registered unemployed persons that find the job within a given month often exceeds the number of vacancies registered during this time (Tyrowicz 2014; Nagel 2016).

As a result, access to reliable data on the inflow of vacancies was the major problem for us as several sources confirmed the poor quality of registered data on vacancies in Poland. In fact, the only plausible quantitative data could be accessed through internet job portals and there was no common database that could be accessed. The solution is API interface that could extract and aggregate the data on job vacancies from the largest job portals in Poland. The other argument that supports the approach is that the role of the Internet in the job-seeking procedure is incontestable all over the world (Suvankulov et al. 2012; Smith and Page 2015, Eurostat, 2018). Nowadays, the internet traffic is still rising and there is strong evidence that the largest flows of job vacancies can be accessed through the network. Such job offers appear on the web portals that aim at pairing job seekers with employers.

**Fig. 2:**
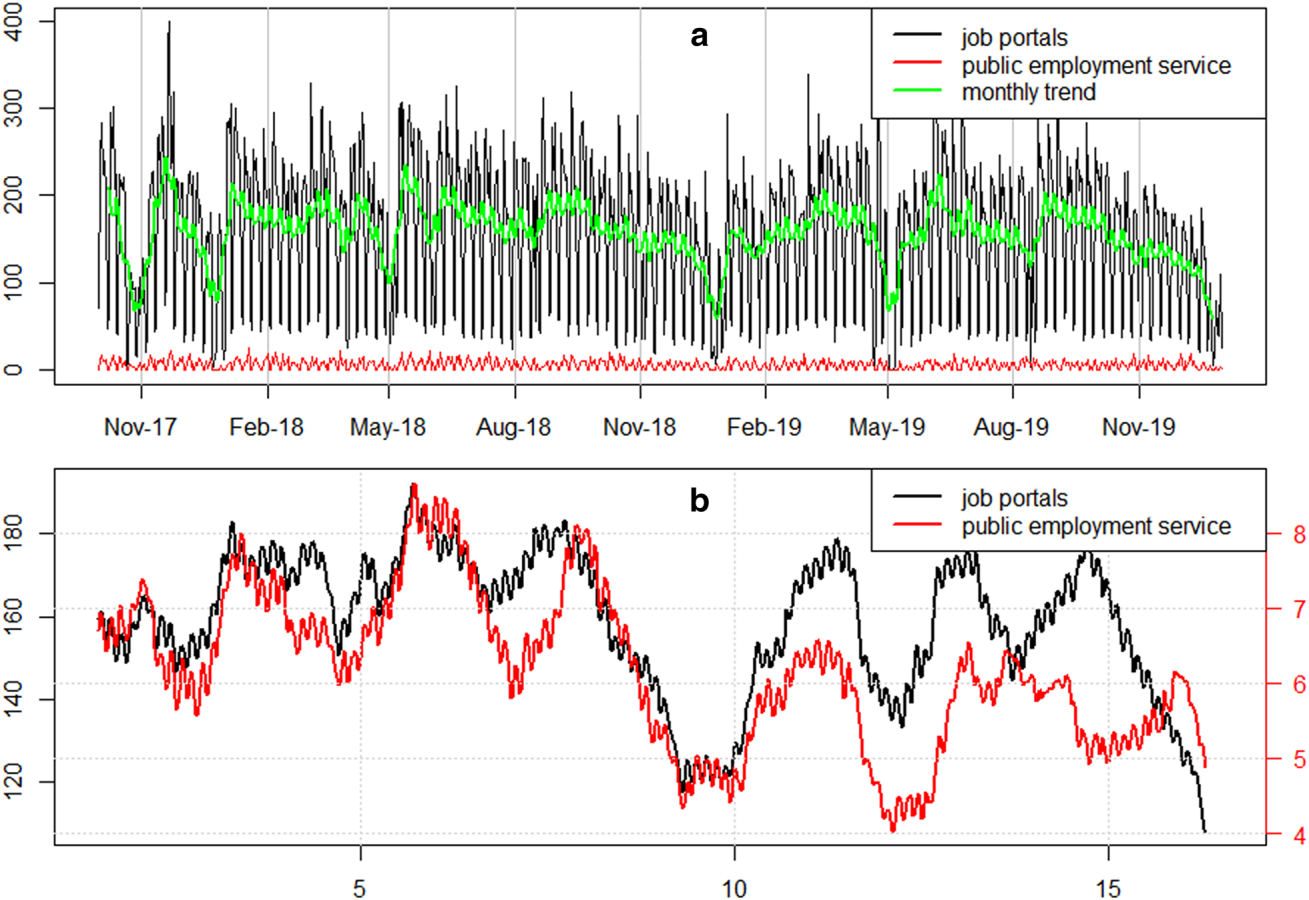
Total daily inflow of vacant jobs in the Poznań agglomeration between 1 October 2017 and 31 December 2019 (**a**) and weekly trend (**b**)*.*commercial job portals are compared with Pubic Employment Service data. The trend for PES data on (B) is shown on the right axis (Source: own elaboration based on the data collected with the API interface from commercial job portals and public employment service. Monthly trends were extracted with classical seasonal decomposition by moving averages (Kendall and Stuart 1983); yearly frequency of time series was set to 12)

Three of the largest Polish internet job offering portals agreed to share their databases with us and produced unique Application Programing Interfaces (API) for our purposes. We started collaboration with the largest Polish job offering portal—http://www.olx.pl (61% of the total market share) as well as the third largest http://www.infopraca.pl (5% of the total market share) according to the Gemius/PBI survey (2019). Additionally, we also received support from one of the smaller players—http://www.jobs.pl (no data on the market share). As we want to compare commercial data with Public Employment Service statistics, we also got API access to the local database of the PES in Poznań. We started developing the API interface and tests in the late 2017, however it reached its full functionality in 2018. In Fig. 2 we present the comparison of the inflow of vacancies since the beginning of the API service operation.

As an example, we plotted registered vacancies (PES) against those downloaded directly from the databases of job portals. It is easy to notice that the difference is enormous and the number of job offers is on average 20 times larger for commercial job portals. In general, massive fluctuations of vacancies are observed with peaks during spring/summer and drops in autumn/winter. A decrease in the number of vacancies is particularly observed in two periods of the year: the beginning of May (short spring holidays in Poland) and Christmas/New Year time. Peaks are mainly observed during spring (March) and early summer (June). When we look into extracted trends from public and commercial data, one can observe some similar patterns in the inflow of vacancies, i.e., the rise and fall in certain periods of the years. However, in general, drops are more severe and peaks are not so dynamic in public data.

### Microdata—a closer look

**Fig. 3:**
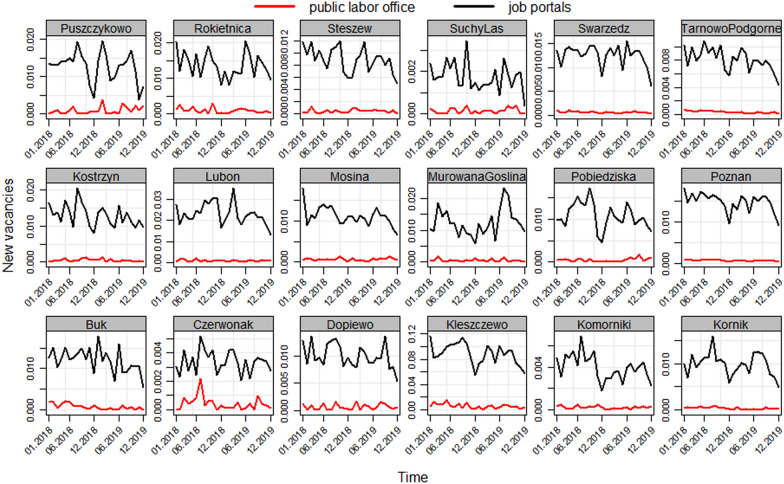
Monthly rates of vacancies* for 18 counties of the Poznań agglomeration in January 2018–December 2019. *vacancies rates (vr) were computed according to the formula: $\mathrm{vr}=\frac{\mathrm{v}}{\mathrm{v}+\mathrm{e}}$, where v is the number of vacancies and e is the number of employed persons. The number of the employed was extracted from the Local Data Bank of CSO at: https://bdl.stat.gov.pl/BDL/start (Source: own elaboration)

The API interface was fully operating in the late 2018, however we managed to extract data starting from the beginning of the year. Then, we disaggregated the data by counties of origin by the field named “location” that indicates the geolocation of each job offer. As a result, vacancies were sorted according to the data source (public vs commercial) and its geographical location. We downloaded data from 01 January 2018 to 31 December 2019 on a daily basis. Every 24 h the API interface connected to the three selected web services and downloaded the data on job vacancies to the SQL database. In addition, also PES data were downloaded every day, however, the daily dynamics of PES data on the inflow of vacancies at county level was very low. In fact, some days with the NULL inflow appeared. The monthly inflow rate of new vacancies is presented in Fig. 3.

**Fig. 4:**
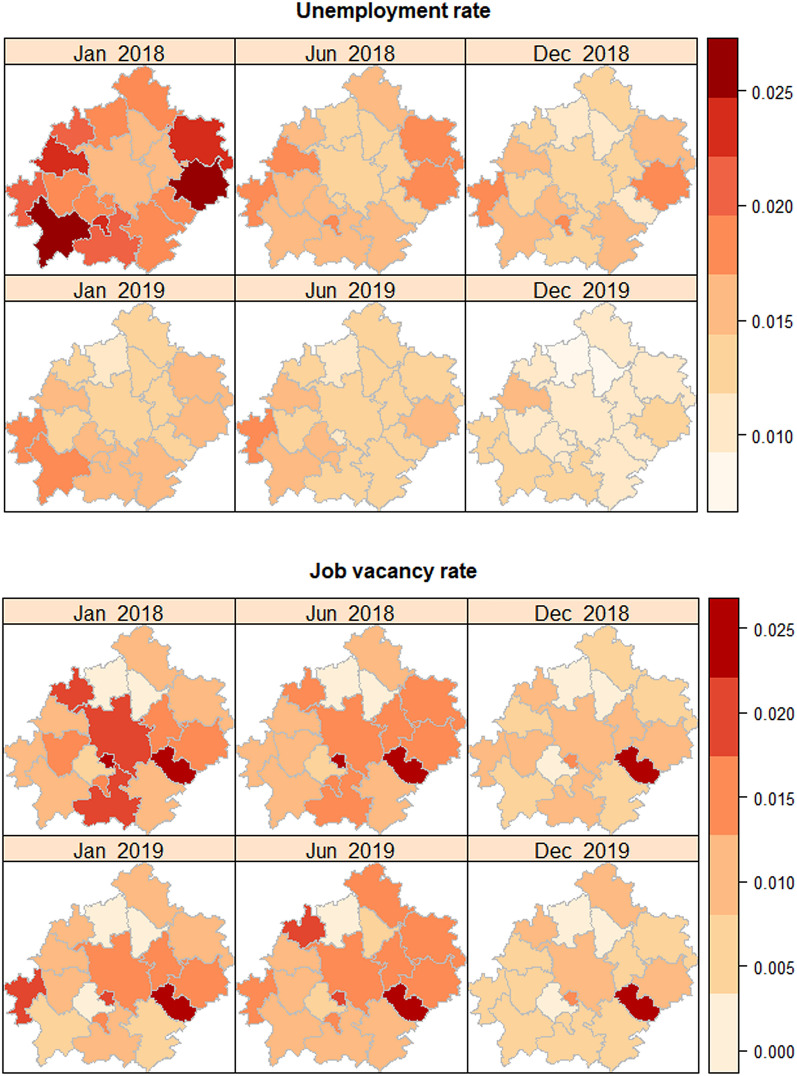
Evolution of the unemployment rate and job vacancy rate in 18 counties of the Poznań agglomeration in 2018–2019*. *unemployment rates were computed on the basis of data reported by PES; job vacancy rates were computed on the basis of data collected from commercial job portals (Source: own elaboration)

In turn, the data on unemployment were extracted from the Public Employment Service (local labor office). The unemployment rate is published by PES monthly for the agglomeration in total. However, we can easily estimate monthly unemployment rate^3^ data for each of 18 counties having the number of the unemployed for each month^4^ and the working-age population for counties derived from Census data.^5^ As one can easily observe (Fig. 4), the unemployment rate is diversified although ultimately low (even below 1% in two counties in December 2019). There are some areas with a slightly higher unemployment rate (southwestern part of the agglomeration) and slightly lower (central and northeastern counties).

For further analysis, we aggregated (summed) the daily vacancies inflow data to monthly frequency to match the registered data on unemployment that are reported monthly. Luckily, in Poland unemployment data are much more reliable, because every person that loses a job has to register in the employment office to get free health insurance. Therefore, in practice a vast majority of unemployed persons register in the labor office to get access to medical services.

Unemployment rate and job vacancies rate dropped during 2018–2019. On average, in January 2018 one can observe higher rates than in December 2019. The last month of the 2019 had a record-low unemployment rate (1.1%) together with 1% vacancies ratio. In turn, in January 2018 the unemployment rate was about 2% accompanied with a similar level of the job vacancy rate. The phenomena led to serious problems related to finding workers and caused, *inter alia*, a significant rise in wages and a massive inflow of migrants from Eastern Europe.

The core metropolitan unit (Poznań) is in the above-average group in terms of vacancies and below-average in terms of unemployment. In general, counties with higher unemployment are located in the peripheries of the agglomeration and counties with lower unemployment are located in the central part of the area.

What is interesting is the case of the Kleszczewo county (a small east-central unit), characterized by constant and the highest job vacancy rate (above 2.5%) and rather permanent unemployment (1.2%). Kleszczewo is a rural county with the lowest number of employees in the agglomeration (989 people in 2018). The agricultural nature of the commune and the above-average inflow of new vacancies (due to the localization of the large distribution center) seem to be the best explanation for the presented indicator level.

When we compute the cross-correlation coefficient for the data, we obtain some puzzling results. The inflow of vacancies is positively correlated with the unemployment inflow. It means that if the inflow towards unemployment is higher, employers are more likely to create new jobs and post vacancies. In turn, if the unemployment inflow is lower, employers are less likely to post new vacancies. This can be explained by the greater willingness of employers to hire workers during higher supply of labor. Both inflows of vacancies are highly correlated (0.79) which means that public and commercial data share some common trend also regarding a given spatial unit. If unemployment is higher, people tend to find jobs more often and the inflow to unemployment also rises (Table 1).

**Table 1:** Cross-correlation matrix

	u	vp	vj	inf	out	ur	pvr	jvr
u	**NA**	–	–	–	–	–	–	–
vp	0.98	**NA**	–	–	–	–	–	–
vj	0.97	0.96	**NA**	–	–	–	–	–
inf	0.98	0.97	0.98	**NA**	–	–	–	–
out	0.97	0.97	0.98	0.99	**NA**	–	–	–
ur	− 0.142	− 0.14	− 0.15	− 0.17	− 0.18	**NA**	–	–
pvr	− 0.04	0.015	− 0.02	− 0.04	− 0.04	0.014	**NA**	–
jvr	− 0.02	0.017	0.01	− 0.02	− 0.02	0.02	0.79	**NA**

u is the number of the unemployed, vp is the number of new vacancies in public statistics; vj is the number of new vacancies gathered via the API interface from commercial job portals; inf is the number of newly unemployed persons; out is new hires; ur is the rate of unemployment; pvr and jvr are vacancy inflow rates from public statistics and commercial job portals respectively

The summary of the data used in further computations is presented in Table 2. All the data relate to LAU2 (NUTS5 in former Eurostat nomenclature) administrative regions. It is worth noting that the data are divided into “rates” and “raw data”. In the case of raw data, the range is huge, because the series for central metropolitan area has skewed distribution to the right. In turn, the rates are normally-distributed with close match between the median and the mean. Time series were tested against stationarity. Test statistics is based on the estimation of augmented Dickey-Fuller (ADF) regressions for each variable as presented by Maddala and Wu (1999). All series except for the number of unemployed persons contain a unit root.

**Table 2:** Descriptive statistics of data

	*Min*	*Max*	*Mean*	*Median*	*Std*	*Unit root*
Raw_data						
u	37	7296	404	161	1027	0.36
vp	0	218	12	3	38	0.00
vj	3	4464	270	50	829	0.00
inf	3	1218	67	23	180	0.00
out	2	547	35	12	94	0.00
Rates						
ur	0.0079	0.026	0.0144	0.014	0.003	0.00
pvr	0	0.014	0.0008	0.0004	0.0015	0.00
jvr	0.0004	0.116	0.015	0.011s	0.019	0.00

u is the number of the unemployed, vp is the number of new vacancies in public statistics; vj is the number of new vacancies gathered via the API interface from commercial job portals; inf is the number of new unemployed persons; out is new hires; ur is the rate of unemployment; pvr and jvr are vacancy inflow rates from public statistics and commercial job portals respectively

### Correlation at the low level spatial labor market

The interaction between spatial units in the labor markets were confirmed in several empirical research (e.g. Patacchini and Zenou 2007; Netrdová and Nosek 2020). Spatial correlation can be particularly observed in local labor markets where a significant share of people commutes long distances every day. Such markets are not independent and function in a network of interconnections determined by the flow of workers and job vacancies.

Consequently, spatial weights matrix *W* for 18 counties of the Poznań metropolitan area is one of the key elements of the spatial models. The matrix defines the type of interactions among counties and it is necessary to compute the strength of correlation between neighboring areas. We can distinguish three general types of spatial matrices which can be included in the model specification. They are based on the construction of checker and figure moves; thus we have the Rooks matrix, Bishops matrix, and Queens matrix (Chen 2012). In the case of the Rooks matrix, interaction with horizontal and vertical neighbors is considered; the Bishops matrix includes neighbors lying in diagonal fields, and the Queens matrix captures interactions with all neighboring regions. There are many more types of spatial weight matrices: geographical (distance or based on travel time), socio-economic (based on relative trade, commuting flows, or similar measures), based on the order of neighbors or simply across borders (Vega and Elhorst 2015). The spatial weight matrix is also one of the most criticized elements of econometric models because it is pre-defined and the estimation procedure does not influence the matrix weights (Arbia and Fingleton 2008). However, there is no ideal way to account for a spatial spillover in the model.

The spatial weights matrix W is a N × N positive matrix that summarizes spatial relations between units. Data appear both in rows and columns. Hence, the non-zero elements of the matrix indicate whether two locations are neighbors. As a consequence, element ${w}_{ij}$ indicates the intensity of the relationship between cross sectional units *i* and *j*. By convention ${w}_{ij}=0$ for the diagonal elements of W. Each element of the matrix is defined as:

1 $$W=\left(\begin{array}{cccc}{w}_{11}& {w}_{12}& \cdots & {w}_{1n}\\ {w}_{21}& {w}_{22}& \dots & {w}_{2n}\\ \vdots & :& \ddots & \vdots \\ {w}_{n1}& {w}_{n2}& \cdots & {w}_{nn}\end{array}\right)$$

N(i) is the vector of neighbors of location *j*, which is determined by the matrix construction (Rooks, Bishops or Queens). The intensities of spatial relations are defined by some preset rules that are usually based on contiguity or distance (Anselin 2001; Getis and Aldstadt 2010). The choice of the matrix used in computations is often quite arbitrary (Vega and Elhorst 2013). To incorporate the spatial information into the model, we construct different spatial weights matrices—we developed three common types of spatial matrices that were included in our models. The first was a neighbors-based Queens style contiguity matrix, thus interactions with all neighbors are defined. The second was distance-based matrix that included three nearest spatial locations for each centroid. The last matrix (a hybrid of distance approach) was graph-based, where the neighbor relationships are defined by the triangulation, which extends outwards to the convex hull of the points and which is planar (as defined in *spdep R* package by Bivand (2018)).

The Queens matrix assumes that workers freely roam the local labor market with no preferences on the localization of job vacancies. In turn, distance-based and graph-based matrices assume that workers prefer jobs which are closer to where they live. The first type of spatial relations may involve higher transactional costs (time, money, etc.) which however may be compensated by e.g. a higher salary. In turn, the second type has lower costs which may be preferable in the case of having a low-paid job or other obligations (e.g. children, family, etc.).

As usual, the matrices were row-standardized in the [0,1] range:

$${w}_{ij}^{s}=\frac{{w}_{ij}}{\sum_{j}{w}_{ij}}$$

**Fig. 5:**
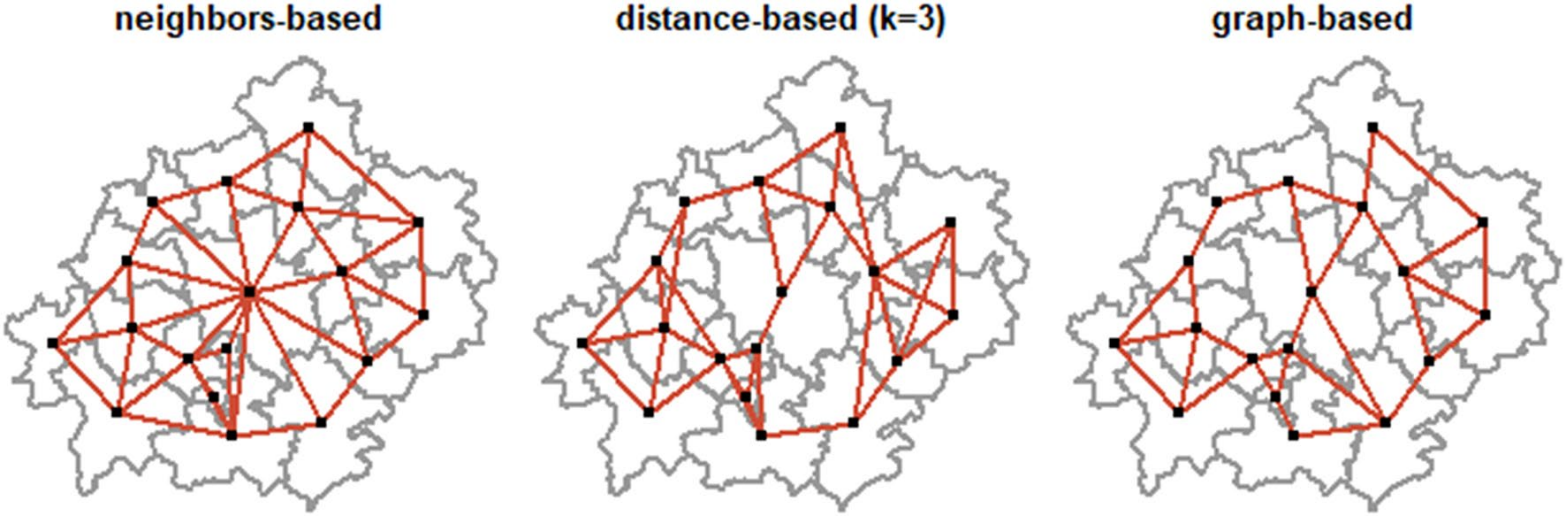
Spatial weights matrices used in computations (Source: own elaboration)

Standardization ensured that the models with different W could be comparable. Finally, we plotted matrices on maps to highlight the differences between them (Fig. 5.

Having the matrices, we could test for spatial autocorrelation. We used two common procedures for calculating the strength of spatial dependencies in the Poznań agglomeration. Moran’s I measure and Geary’s C test. We use the Monte Carlo simulation approach for computing both: Moran’s and Geary’s statistics. MC proved statistics effectiveness (Ren et al. 2014). In that case values are randomly assigned to the polygons, and the measures of spatial correlation are computed. This is repeated several times to establish the distribution of expected values. The observed values (Moran’s I and Geary’s C) are then compared with the simulated distribution to see how likely it is that they do not co-vary. The results of Moran’s I and Geary’s C tests for the unemployment rate and vacancies ratio are reported in Table 3.

**Table 3:** The Moran and Geary tests for spatial correlation

Matrix	ur		jvr		pvr	
	Moran I	Geary C	Moran I	Geary C	Moran I	Geary C
Contiguity	0.16 *(0.04)*	0.87 *(0.21)*	− 0.04 *(0.3)*	0.79 *(0.15)*	− 0.005 *(0.12)*	0.77 *(0.1)*
Distance-based	0.06 *(0.09)*	0.83 *(0.07)*	0.04 *(0.04)*	0.82 *(0.11)*	0.03 *(0.04)*	0.78 *(0.12)*
Graph-based	0.07 *(0.21)*	0.86 *(0.06)*	− 0.04 *(0.33)*	0.86 *(0.12)*	− 0.01 *(0.15)*	0.82 *(0.08)*

ur is the rate of unemployment; pvr and jvr are vacancy rates from public statistics and commercial job portals respectively

Similarly, as Galecka et al. (2017) proved, spatial interactions are stronger for stock than for inflows^6^ in the case of Poland. The dependency is also visible in our dataset (Table 3)—correlations are significant rather for the unemployment rate than the vacancies inflow rate. According to p-values, Global Moran’s and Geary’s tests show weak positive spatial correlation for the unemployment rate in the Poznań agglomeration. In turn, both job vacancy rates seem to be randomly distributed in space and do not follow spatial patterns. The results, however, strongly depend on the kind of spatial matrix used in computations (e.g. according to Moran’s I computed with a distance-based matrix there is evidence for weak, positive spatial correlation also in the case of both vacancy rates).

**Fig. 6:**
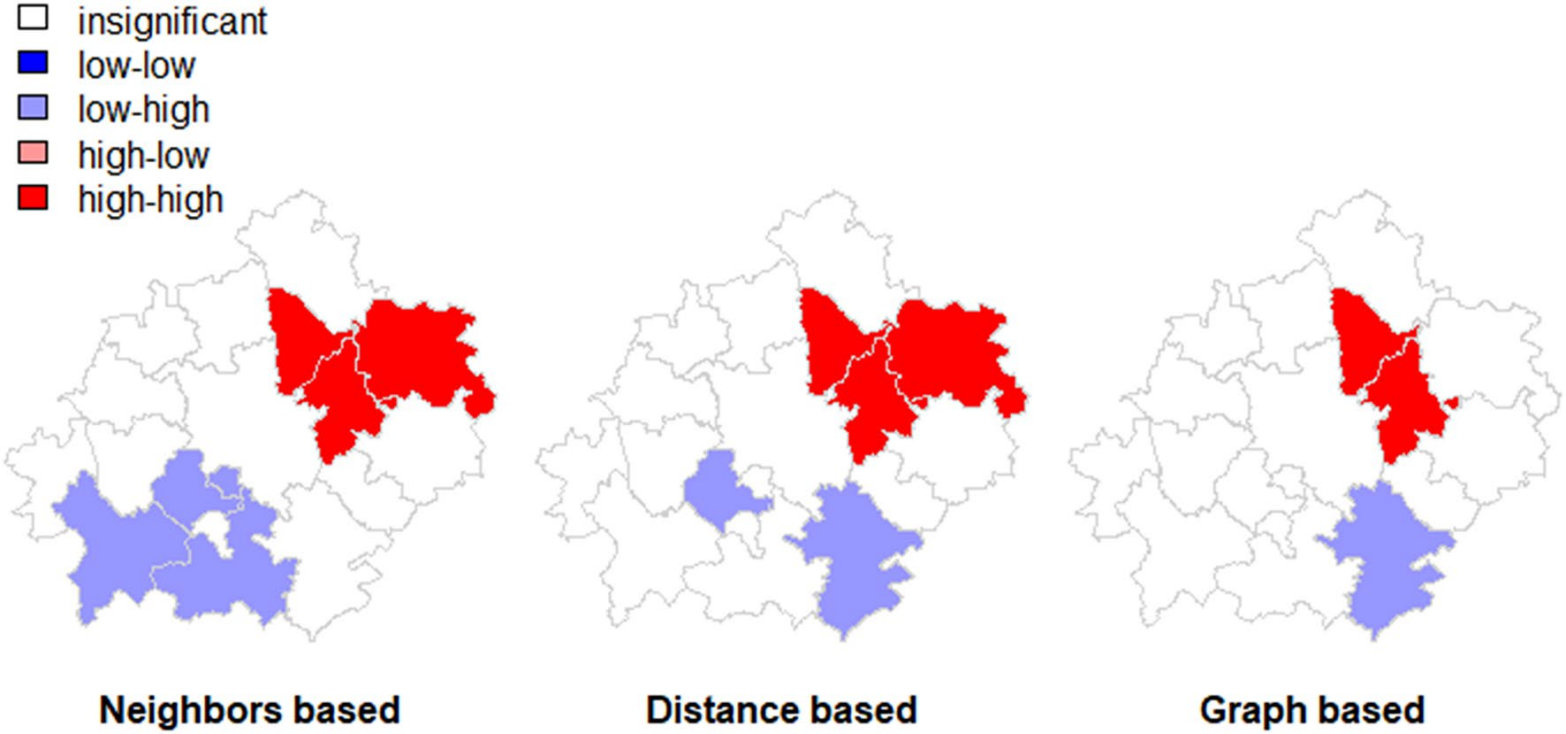
LISA clusters for the unemployment rate (mean value for 2019, local Moran statistics) ( Source: own elaboration)

As global tests are calculated from local relationships, we moved to local indicators of spatial correlation (LISA) that measure the correlation between the same variables in the two neighboring spatial units (Anselin 1995). Two measures are usually employed for clustering: local Moran and Getis-Ord statistics. Such indicators detect clusters of similar values around a particular location or identify areas that do not follow the global trend. As the first indicator local Moran statistics was identified for the significance level = 0.1 and plotted on Fig. 6

The strongly colored regions are therefore those that contribute significantly to a positive global spatial autocorrelation outcome, while paler colors contribute significantly to a negative autocorrelation outcome. This means that pale colors are surrounded by dissimilar values that occur near one another. The north-eastern cluster shows significant clustering for the unemployment rate in the agglomeration. On the other hand, the south-western cluster contributes negatively to the autocorrelation outcome and is surrounded by units of dissimilar values. The Getis-Ord Gi statistic (Ord and Getis 1995) looks at neighbors within a defined proximity to identify where either high or low values cluster spatially (Fig. 5). Extracted clusters are areas with significant z-values.

### Spatial panel matching function models

To investigate in-depth the spatial relationship between supply and demand on the local labor market we develop spatial panel matching function models. We also compared the spatial models with its non-spatial equivalents. The general model is based on the labor market matching function by Mortensen and Pissarides (e.g. Mortensen 1986; Mortensen and Pissarides 1994; Pissarides 2000).

**Fig. 7:**
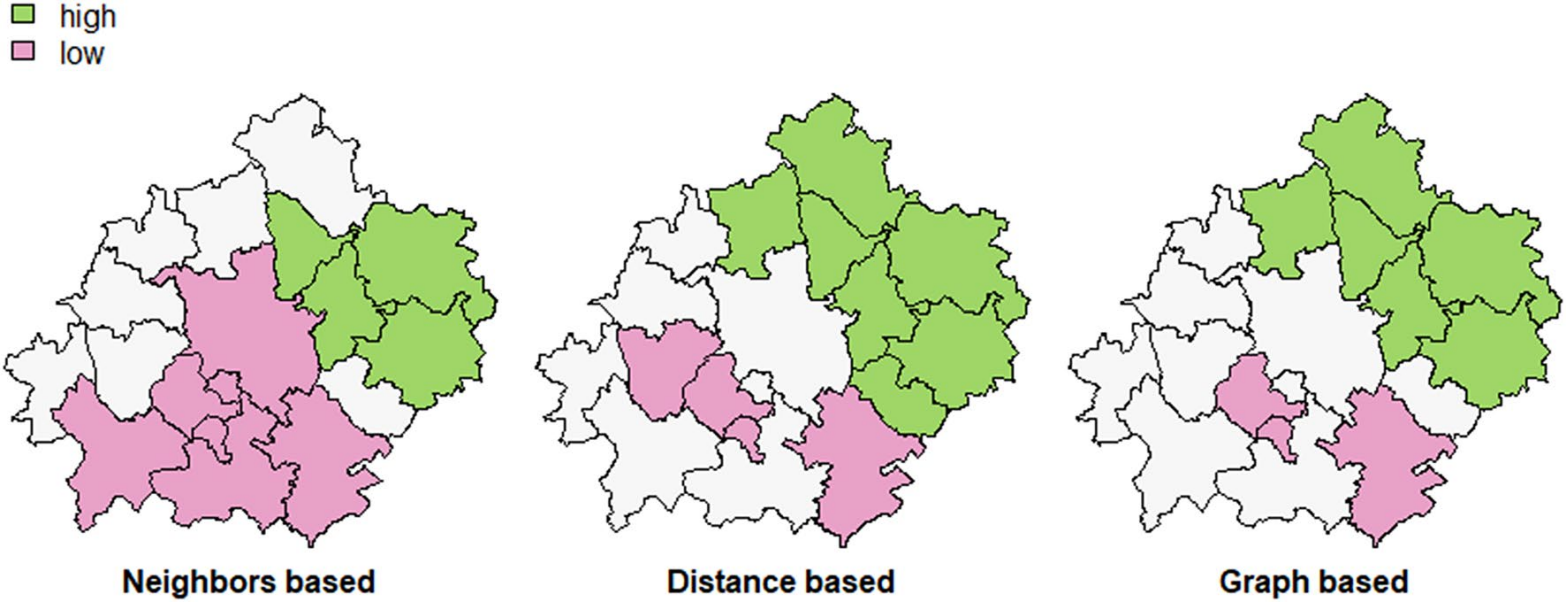
LISA clusters for the unemployment rate (mean value for 2019, Gettis-Ord statistics) (Source: own elaboration)

As we noticed in the previous section, the role of spatial interactions in the local labor market is undoubtful. The number of new vacancies is not evenly distributed in space and force workers to commute. Daily commuting is a widely spread phenomenon in almost every city agglomeration (e.g. Wong et al. 2020). It was also investigated and confirmed for Poznań and surrounding counties by e.g. Bul (2014). Job seekers may search and match vacancies in different counties if they find a job that suits them best (a better job or a higher salary). Parallelly, employers hire workers from different geographical locations as they are interested rather in skill match that does not depend on geolocation. These spatial aspects of the matching process are reflected in the model that we present in the further elaboration (Fig. 7).

The matching function governs how agents meet with each other in a spatial environment. The function captures search frictions that occur during the process and produces the number of matches having the given arguments. The number of new matches $M$ on the labor market is the result of two inputs: unemployment stock $U$ and vacancies stock $V$ ($M=m\left(U,V\right)$). Further developments of this basic model consider a so-called flow approach in which besides stocks we include flows of unemployed and vacancies: $M=m\left(U,V, u, v\right)$ (Blanchard and Diamond 1994). We can also distinguish mixed models that include values of a single stock or flows: $M=m\left(U, v\right)$ (Gałecka-Burdziak 2017). These models can be useful in modeling the job search process when we can easily assume that stock of unemployed trade with the inflow of new vacancies.

In the paper we present the combination of the stock with flow model.^7^ Therefore, new vacancies trade with both: unemployed persons (U) and the inflow of the newly registered unemployed (u). Consequently, the general specification of the matching function can be written as:

$$M=m\left(U, u, v\right).$$

The economic foundation of this approach is provided by, e.g. Coles and Petrolongo (2008). A laid-off worker seeks for a new vacancy as quickly as possible. If the worker is lucky, he or she can quickly exit unemployment, even during the same month. Also, the approach that the unemployed trade rather with the inflow than with the stock of vacancies is supported by empirical research—studies suggest that the vast majority of newly posted vacancies is filled during the first 7 days (e.g. Burdett and Cunningham 1998). Job seekers tend to scroll new vacancies; they hardly ever go back to the older job adds.

The situation corresponds to the conditions on the local labor market in Poznań. A record-breaking low unemployment rate resulted in a persistent unemployment stock which consists mainly of those who are unable or unmotivated to start legal employment. As a result, the employers trade rather with the inflow of unemployed persons than with the stock. In that case, a majority of new hires is connected with employee turnover, frictional unemployment or the inflow of workers from other regions or countries (e.g. Ukraine or Belarus).

Consequently, the model can be written as a common Cobb-Douglass formula: $M=A{\mathrm{U}}_{T}^{\alpha }{v}^{\beta }$. Note that the stock-flow implies that the total number of unemployed U_T_ can be decomposed into the stock of unemployment *U* as well as the inflow of newly registered persons *u*. The model assumptions are that $\frac{\partial M}{U}>0,\frac{\partial v}{U}>0$ and $\frac{\partial {M}^{2}}{U}<0, \frac{\partial {M}^{2}}{v}<0$ so the function is increasing in both arguments but its increments are decreasing.

Further, however a questionable assumption is the one about constant returns to scale of the function. The latter was either falsified or confirmed in several empirical studies (e.g. Shimmer (2005); Guerrazzi (2015)) and it is also a point of our interest regarding the low-level of spatial aggregation. Therefore, we can simplify further calculations and write the model in the logarithmic form of a linear model:

2 $$logM=logA+{\alpha }_{1}log(U)+{\alpha }_{2}log(u)+\beta log(v)$$

Further, the model is enhanced with spatial dependencies according to the distance-based spatial weights matrix. We use the matrix because it provided the highest average estimates for spatial correlation for all variables (Table 3).

The spatial dependencies can be included in the model in several ways: by a spatial lag of either a dependent or independent variable. The error component can be also enhanced by spatial spillovers (Anselin et al. 2008). To keep things clear, we make use of spatial econometric models’ taxonomy provided by Vega and Elhorst (2013; 2015). On its basis we develop three different spatial matching function models in terms of spillover and direct effect. We take the model with spatial lags of independent variables (SLX) as a point of departure as suggested by Vega and Elhorst (2015). SLX allows accounting for both direct and spillover effects and is a complement of the critique of spatial econometrics. In terms of (2), the SLX panel model for a cross-section of N observations over time $t=1\dots T$ may be written as:

3 $${Y}_{t}=\alpha {l}_{N}+{X}_{t}\beta +W{X}_{t}\theta +{\varepsilon }_{t}, {\varepsilon }_{t}\sim \left(0,{\sigma }^{2}{l}_{N}\right),$$

where $Y$ is the vector dependent variable; X is the matrix of explanatory variables; W is a positive N × N matrix; $\alpha$ is the parameter of constant term; $\beta ,\theta$ are the vectors of model parameters. Rewriting (3) of the empirical case presented in this study comes to the SLX matching function panel model:

4 $$\mathit{log}\left({M}_{i,t}\right)=\alpha +{\beta }_{1}\mathit{log}\left({U}_{i,t}\right)+{\beta }_{2}\mathit{log}\left({u}_{i,t}\right)+{\beta }_{3}\mathit{log}\left({v}_{i,t}\right)+{\theta }_{1}{W}_{i,j}\mathit{log}\left({U}_{i,t}\right)+{\theta }_{2}{W}_{i,j}\mathit{log}\left({u}_{i,t}\right)+{\theta }_{3}{W}_{i,j}\mathit{log}\left({v}_{i,t}\right){+ \varepsilon }_{i,t}$$

In that case, the direct effects are the coefficient estimates of nonspatial variables $\beta$ and the spillover effects are those associated with the spatially lagged explanatory variables $\theta$; *i, j* are spatial indexes and *t* is the time index. SLX includes spatial interactions of explanatory variables so we can assess the impact of spatially lagged exogenous variables on the number of new matches. However, several econometric studies investigate also endogenous interaction effects or interactions between error terms (e.g. Lee and Yu 2016). The nature of spatial interactions on the labor market is complex and results from different factors which are sometimes difficult to identify (Vega and Elhorst 2016).

Having this in mind, the second model is the spatial Durbin error model (SDEM) which is the extension of the SLX model and can also be useful in detecting local spillovers. Additionally, the model assumes the spatial pattern in an error term due to omitted factors. The SDEM can overcome both spatial autocorrelation relationships of independent variables and spatial errors between regions. Formally, SDEM is enhanced SLX and contains both spatial exogenous interaction effects as well as a spatial error term. Thus, ${\varepsilon }_{i,t}$ in (4) evolves to $\lambda {W}_{i,j}\varepsilon +u$, where $\varepsilon$ is a specific space effect term that can be either correlated or not with the regressor,$\lambda$ is the parameter describing the strength of spatial correlation among errors and $\mathrm{u}=N(0, {\sigma }^{2})$ is white noise. SDEM however fails in the identification of global spillover effects which can be also significant on the labor market.

Consequently, the third specification of a spatial model is the spatial autoregressive model (SAR). It includes the spatial lag of a dependent variable and allows detecting global spatial effects. In that case, including endogenous interaction effects means that the number of new matches in a given unit may affect other units in the agglomeration even if the units do not have the common borders. It could be justified economically as unemployed persons seek for a job in the whole agglomeration and may commute throughout several counties. Job search and matching is not limited to neighboring units. Formally speaking the spatial matching function SAR model is:

5 $$\mathit{log}\left({M}_{i,t}\right)=\alpha + \delta {W}_{i,j}log\left({M}_{i,t}\right)+{\beta }_{1}\mathit{log}\left({U}_{i,t}\right)+{\beta }_{2}\mathit{log}\left({u}_{i,t}\right)+{\beta }_{3}\mathit{log}\left({v}_{i,t}\right){+ \varepsilon }_{i,t},$$

where $\delta$ is the spatial coefficient. In turn, the common panel model without a spatial component would turn into:

6 $${log(M}_{i.t})=\alpha log{(A}_{i,t})+{\beta }_{1}log\left({U}_{i,t}\right)+{\beta }_{2}log\left({u}_{i,t}\right)+{\beta }_{3}log\left({v}_{i,t}\right)+{\mu }_{i}+{e}_{i,t}.$$

In further computations we used the data described in the previous section. Thus, we had a balanced panel with n = 18, T = 24 and N = 432. Due to the limitations of the API interface, we were able to retrieve 24 months’ time-series for each panel unit which could be a little too short (e.g. Griffith 2013). We are fully conscious of the limitations of the data, however, some empirical studies successfully use even smaller datasets. For instance, Pereira et al. (2017) developed a spatial regression model for 28 Portuguese NUTS3 and 12 time points (N = 336). The authors argue that small area estimation methods, "borrow strength" from adjacent regions and therefore compensate for the small sample sizes which is often observed in these areas. In turn, Acosta and Valejos (2018) provide a simulation of information loss depending on the n in spatial regression. They show that including 25 units provides the evidence for spatial correlation only slightly weaker than including 50 units. They also conclude that strong prior information may compensate for a small sample size.

The spatial models were tested and confirmed for individual effects with the Lagrange Multiplier test for spatial panel models. Time fixed effects proved to be nonsignificant in an overall test (Baltagi et al. 2003). The fixed effects estimator was chosen on the basis of a Hausman spatial panel models test extension (Mutl and Pfaffermayr 2011; Bell et al. 2019). The tests results are reported in Tables 4 and 5.

**Table 4:** Estimation results for spatial panel and panel models with commercial job portals data on vacancies*

	Spatial panel models**			Non-spatial panel models	
Variable	SAR	SLX	SDEM	Fixed effects	Pooled
A	–	–	–	–	− 1.49*** *(0.000)*
Wlog(M)	0.23*** *(0.000)*	–		–	–
Log(u)	− 0.18 *(0.11)*	− 0.37 *(0.11)*	− 0.37 *(0.07)*	− 0.20 (0.10)	0.42*** (0.000)
log(inf)	0.169* *(0.017)*	0.177* *(0.02)*	0.174* *(0.015)*	0.19* (0.01)	0.53*** (0.000)
log(v)	0.22*** *(0.005)*	0.20* *(0.01)*	0.21** *(0.003)*	0.25*** *(0.000)*	0.04* *(0.1)*
Wlog(U)	–	0.14 *(0.57)*	0.18 *(0.44)*	–	–
Wlog(inf)	–	0.13 *(0.24)*	0.11 *(0.36)*	–	–
Wlog(v)	–	0.12 *(0.24)*	0.07 *(0.47)*	–	–
We	–		0.22*** *(0.000)*	–	–
R-squared	0.88	0.06	0.87	0.05	0.84
Spatial Hausman	7.95 *(0.04)*	119.78 *(0.000)*	45.4 *(0.000)*	94.29 *(0.000)*	–
LM test for effects***	5.27 *(0.000)*	0.92 *(0.07)*	4.70 *(0.000)*	–	–
AIC/BIC	378/472	397/498	283/489	395/484	444/460

*p-values are reported in brackets

** Spatial models were estimated with region fixed effects.

***LM is the Lagrange Multiplier test for individual effects

**Table 5:** Estimation results for spatial panel and panel models with public employment service data on vacancies*

	Spatial panel models**			**Non-spatial panel models**	
Variable	**SAR**	**SLX**	**SDEM**	**Fixed effects**	**Pooled**
A	–	–	–	–	1.41*** *(0.000)*
Wlog(M)	0.24*** *(0.000)*	–		–	–
Log(u)	-0.10 *(0.38)*	-0.34 *(0.14)*	-0.38 *(0.1)*	-0.10 (0.39)	0.44*** (0.000)
log(inf)	0.184** *(0.009)*	0.191* *(0.01)*	0.178* *(0.014)*	0.20** (0.006)	0.54*** (0.000)
log(v)	0.02 *(0.41)*	0.02 *(0.46)*	0.04 *(0.35)*	0.03 *(0.26)*	0.04 *(0.08)*
Wlog(U)	–	0.23 *(0.38)*	0.29 *(0.23)*	–	–
Wlog(inf)	–	0.18 *(0.12)*	0.10 *(0.35)*	–	–
Wlog(v)	–	0.05 *(0.29)*	0.04 *(0.35)*	–	–
${\varvec{\lambda}}$/We	–		0.23*** *(0.000)*	–	–
R-squared	0.87	0.03	0.88	0.02	0.84
Spatial Hausman	94 *(0.000)*	67.5 *(0.000)*	80.31 *(0.000)*	74.13 *(0.000)*	–
LM test for effects***	6.55 *(0.000)*	1.22 *(0.1)*	6.26 *(0.000)*	–	–
AIC/BIC	389/483	397/498	392/497	408/497	444/460

*p-values are reported in brackets

** Spatial models were estimated with region fixed effects, ***LM is the Lagrange Multiplier test for individual effects

Finally, we have estimated 10 models on the basis of formula (4), (5) and (6). Five models were estimated with job portals data on vacancies and other five with PES data on job vacancies. For each of these two groups three models were spatial panels (SLX, SDEM and SAR) and two models were regular panels without spatially lagged variables (a fixed-effect estimator and a pooled model). We used a distance-based matrix in computations as it has proven the highest values for spatial correlation (on average).

Spatial models were estimated with two-step Maximum Likelihood as implemented in*splm* R package^8^ (Millo and Piras 2012).^9^ In turn, the regular panel model was estimated with a fixed effect estimator; a pooled model was assessed with an OLS approach. In that case we used *plm* R packge^10^ (Croissant and Millo 2018) that provides a set of estimators for panel data.^11^ The results are presented in Table 4 (commercial data on job vacancies) and Table 5 (PES data on job vacancies).

Comparison of both R-squared and AIC/BIC criteria leads to the conclusion that spatial models estimated with commercial job ads are better fitted with the data (they produced higher R-squared and lower AIC/BIC scores). In turn, spatial models estimated with PES data have not confirmed the existence of the matching function. The inflow of new vacancies is not significant in that case; thus, we focus on the analysis of the output of models estimated with combined data (PES data on unemployment + commercial job portals data on vacancies).

Therefore, the SAR, SLX, SDEM and panel fixed-effects models identified the spatial matching function which exhibits strictly decreasing returns to scale. In turn, the pooled model indicates the constant return to scale matching function. The result corresponds to the papers that estimate the matching function with disaggregated data (e.g. Yashiv (2006) or Kangasharju et al. (2005)). We can than support a hypothesis that some studies with the exemplary well-known paper by Petrolongo and Pissarides (2001) may overstate the dominance of the CRS matching function.

Spatial panel models show that matching technology on the market is the result of trade between the inflow of new vacancies and the inflow of unemployed persons rather than the stock which appeared insignificant. The coefficient of the unemployment inflow was a little lower than the coefficient of the inflow of vacancies. It means that an increasing inflow of unemployment and vacancies by 1% would result in an increase in new matches by ~ 0.17% and ~ 0.21% respectively. This finding may reflect the situation on the local labor market. Ultimately, the low unemployment rate of 1.1% and a high demand for workers force the employers to trade with the inflow of the newly unemployed rather than with the stock that consists of persons that are not well-motivated or unable to start work. Therefore, new matches result from worker turnover, migrants from other regions and countries, and those entering the labor market from the inactivity zone.

The SAR model generates the strong process of global spillovers showing that the effect was observed in the study area. It indicates that if the number of new matches (*M*) changes in one unit, it somehow affects all units in the agglomeration. $Wlog(M)$ is a spatially lagged dependent variable the coefficient of which is highly significant and positive. It appears that the matching process in one region significantly affects the outflow in other areas with positive externalities. The larger the outflow in a given area, the larger in the rest of the units. It may be the result of the great mobility of workers on the urban labor market that commute long distances (through several counties) to get to work. Thus, workers in the agglomeration (globally) may match with vacancies that appear in a given county (locally).

The SLX and SDEM models indicate that local spillover effects are not significant. Local spillovers mean that a given region affects the adjacent units while not having effect on the rest of counties. This finding supports the existence of global effects on the local labor market. In the case of SDEM, the additional variable is the spatially lagged error term (${\varvec{\lambda}}$/We) that is positive and significant. ${\varvec{\lambda}}$ estimates indicated that the shock in a given region may be induced by unobservable effects in adjacent areas. Thus, SDEM provides the evidence of spatial autocorrelation because of other significant factors that were omitted in the specification of the matching function. This finding, in turn, may partially explain the decreasing return to scale matching function.

## Conclusions

The aim of this paper was to investigate spatial processes on low-level spatial labor markets with a unique set of data. We extracted data on job vacancies from the three largest commercial job portals in Poland. Unlike other studies that use public statistics, we employed a dedicated API-based service to download and aggregate the data across spatial units to obtain more a reliable insight into demand for workers. Our analysis focused on the one of the largest urban areas in Poland (Poznań agglomeration) that consists of 17 small spatial units of the LAU2 class. We proved the existence of spatial autocorrelation and identified the clusters that significantly contribute positively and negatively to the unemployment rate. We therefore extend studies on labor market spatial correlation by adding the analysis of small spatial units which have not been the object of studies so far. We also contribute by the analysis of the spatial panel matching function on the urban labor market with a unique dataset combined with the registered data on unemployment and commercial data on job adds.

In the paper, we estimated spatial and non-spatial models with public and commercial data on job vacancies. The results clearly show the drawbacks of PES data on vacancies. Although estimations proved the existence of spatial dependencies, the PES estimations did not identify the matching function and produced worse fit to data. In effect, we were able to produce further conclusions on the basis of the models estimated with the data on job vacancies extracted from commercial job portals. In addition, if we were only to follow the estimates of the pooled models, we would confirm the common finding of the existence of the CRS matching function also at the low level of spatial aggregation (as identified by both pooled models). However, the estimated spatial models exhibit decreasing returns to scale (DRS) and this finding goes along with the hypothesis of exaggerating the role of the CRS matching function (e.g. Yashiv (2006) or Kangasharju et al. (2005)).

We believe, DRS in that case are mainly the result of the omitted variables (as identified by the SDEM). These unidentified factors may be connected with on-the-job search or the exploitation of other channels in the recruitment procedure and bias the elasticities of the matching function ((Fahr and Sunde 2005; Sunde 2007). These additional factors may be particularly important because of permanent worker shortages on the local labor market. One should remember that our analysis was carried out for the specific urban labor market that was characterized by the record-low unemployment level (~ 1.5% on average) and a high demand for workers. In fact, in 2018–2019, workforce shortage was the largest problem on the local labor market (LMO 2020). Moreover, under an extremely low unemployment rate firms are not likely to unthinkingly post new vacancies—a job add is an additional cost which might not return. Hence, the positive sign of cross-correlation between these two variables. As a result, creativity counts and various search behaviors are used in order to find a trading partner.

Another finding (made on the basis of API-gathered data) includes strong positive externalities connected with the matching process among 18 LAU2 units of the urban labor market. The externalities are global in their nature which means the one outflow to employment in one unit affects also others not necessarily the closest ones. The externality may be connected with job commuting—workers may travel through several counties and an increase in the number of new vacancies in one unit attracts job seekers in the whole agglomeration.

On the basis of our study, we could provide two main areas of recommendations. Global spillovers indicate strong economic connections between counties and imply policy solutions to be implemented at global rather than local level. From this point of view, further development of the common transportation system should be the primary objective. This kind of solution may reduce matching frictions and transactional costs and increase the overall performance of the local labor. Also, a special point of interest should be solutions for foreign workers, especially from Ukraine or Belarus, who are already present on the local labor market. The immigrants may help to fill the huge gap in labor supply. However, the complicated and time-consuming institutional procedures may discourage them from looking for a job. The institutional weakness is also reflected in the extremely underestimated data on registered vacancies. PES is not fulfilling its function in the job-workers matching process. Thus, the other recommendation would be improving job placement services and increasing the cooperation between employers and PES.

The present situation connected with the shock of the COVID-19 pandemic did not hit particularly severe the local labor market—the registered unemployment rate in December 2020 in the Poznań agglomeration was still very low and reached merely 2.1%. As a result, the identified issues of the spatial matching process may evolve and should be a point of interest also in the near future.

## Data Availability

The datasets and scripts generated and/or analysed during the current study are available in the GitHub repository, https://github.com/wozniak2/spatialMF.

## Footnotes

1 A type of network-based interface that uses Web architecture and protocols (in particular the HTTP protocol) to directly communicate between applications located on separate devices on the network. In that case we use API to download data from internet job portals databases to one independent server.

2 In Poland but also in the majority of European countries there is no obligation to report job vacancies to public employment services.

3 Unemployment rate for each LAU2 was computed according to formula: the number of *unemployed persons/labor force*.

4 The number of unemployed persons is published monthly by local PES service at: https://poznan.praca.gov.pl/

5 The Census data on population and labor force are available at: https://bdl.stat.gov.pl/BDL/start

6 We have no data on vacancy stocks so we focused on the unemployment rate that confirmed spatial correlation significance.

7 See Shaphiro and Stiglitz (1984) for more details on a job queuing approach. In turn, Ebrahimy and Shimer (2010) provide a theoretical framework for stock-slow matching.

8 The package is available from the Comprehensive R Archive Network at http://CRAN.R-project.org/package=splm

9 The package contains routines to the estimation of various spatial models. It is based on the maximum likelihood implementation of the specifications of models with individual effects that are/are not spatially autocorrelated.

10 The package is available from the Comprehensive R Archive Network at https://cran.r-project.org/web/packages/plm/index.html

11 There are several routines that support estimation of different types of panels (*inter alia,* fixed and random effects, variable coefficient models, the general method of a moments estimator, general feasible generalized least squares models).
